# Regulation of HTLV-1 Tax Stability, Cellular Trafficking and NF-κB Activation by the Ubiquitin-Proteasome Pathway

**DOI:** 10.3390/v6103925

**Published:** 2014-10-23

**Authors:** Alfonso Lavorgna, Edward William Harhaj

**Affiliations:** Department of Oncology, Sidney Kimmel Comprehensive Cancer Center, Johns Hopkins School of Medicine, Baltimore, MD 21287, USA; E-Mail: alavorg1@jhmi.edu

**Keywords:** HTLV-1, Tax, ubiquitin, NF-κB

## Abstract

Human T-cell leukemia virus type 1 (HTLV-1) is a complex retrovirus that infects CD4+ T cells and causes adult T-cell leukemia/lymphoma (ATLL) in 3%–5% of infected individuals after a long latent period. HTLV-1 Tax is a *trans*-activating protein that regulates viral gene expression and also modulates cellular signaling pathways to enhance T-cell proliferation and cell survival. The Tax oncoprotein promotes T-cell transformation, in part via constitutive activation of the NF-κB transcription factor; however, the underlying mechanisms remain unknown. Ubiquitination is a type of post-translational modification that occurs in a three-step enzymatic cascade mediated by E1, E2 and E3 enzymes and regulates protein stability as well as signal transduction, protein trafficking and the DNA damage response. Emerging studies indicate that Tax hijacks the ubiquitin machinery to activate ubiquitin-dependent kinases and downstream NF-κB signaling. Tax interacts with the E2 conjugating enzyme Ubc13 and is conjugated on *C*-terminal lysine residues with lysine 63-linked polyubiquitin chains. Tax K63-linked polyubiquitination may serve as a platform for signaling complexes since this modification is critical for interactions with NEMO and IKK. In addition to NF-κB signaling, mono- and polyubiquitination of Tax also regulate its subcellular trafficking and stability. Here, we review recent advances in the diverse roles of ubiquitin in Tax function and how Tax usurps the ubiquitin-proteasome pathway to promote oncogenesis.

## 1. Introduction

Human T-cell leukemia virus type 1 (HTLV-1) is the etiological agent of adult T-cell leukemia/lymphoma (ATLL) and the neurodegenerative disorder HTLV-1 associated myelopathy/tropical spastic paraparesis. It is estimated that 20 million people worldwide are infected with HTLV-1 with 3%–5% of infected individuals developing ATLL after a prolonged latent period (40–60 years) [1]. There are four recognized clinical subtypes of ATLL: chronic, smoldering, lymphoma-type and acute [2]. Of these distinct entities, lymphoma-type and acute present as highly aggressive disease. Despite modest advances in treatment regimens over the past two decades, ATLL remains an incurable and invariably fatal disease.

HTLV-1 predominantly infects CD4^+^ T lymphocytes *in vivo* although it can also infect dendritic and myeloid cell lineages [3]. HTLV-1 virions are poorly infectious and cell-mediated infection is much more efficient than cell-free infection [4]. The HTLV-1 envelope protein interacts with glucose transporter GLUT-1, heparan sulfate proteoglycans (HSPGs) and neuropilin-1 (NRP1) to facilitate viral entry into cells [5,6,7]. Productively infected HTLV-1+ cells establish a virological synapse by cell-cell contact with uninfected T cells mediated by interactions between ICAM-1 and LFA-1 adhesion molecules [8,9]. The virological synapse mediates the accumulation and spread of HTLV-1 core complexes and the HTLV-1 genome to uninfected T cells.

**Figure 1:**
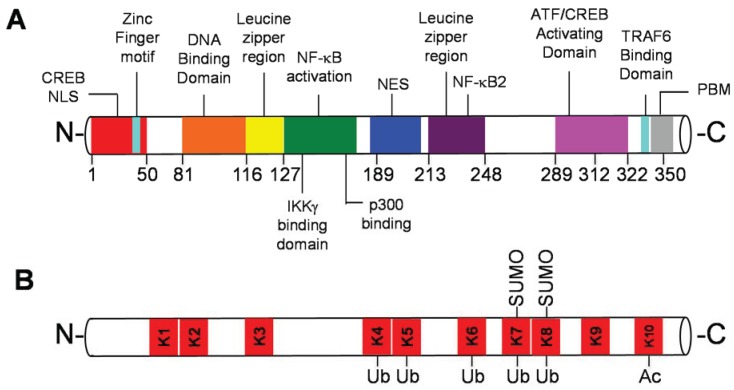
(**A**) Schematic representation of Tax protein-protein interaction domains and other motifs regulating Tax function; (**B**) Schematic representation of known ubiquitination, SUMOylation and acetylation sites in Tax.

The HTLV-1 genome is flanked by 5' and 3' long terminal repeat (LTR) sequences that contain *cis*-acting elements that regulate the expression of viral proteins necessary for the virus to infect and replicate in host cells. Tax is a key HTLV-1 regulatory protein encoded by an open reading frame in the pX region [10]. Tax is a *trans*-activating protein that regulates viral gene expression by recruiting host transcription factors including CREB and coactivators such as CREB binding protein (CBP) to the LTRs [11]. In addition to regulating viral gene expression, Tax also modulates host signaling pathways to induce cell transformation. The oncogenic function of Tax was first elucidated *in vivo* by the generation of Tax transgenic mice which developed distinct tumors depending on the promoter used to drive Tax expression [12,13]. More recently, transgenic expression of Tax in T cells driven by the proximal Lck promoter yielded diffuse large-cell T-cell lymphomas and leukemia resembling acute ATLL [14]. Tax has pleiotropic functions and regulates multiple cellular signaling pathways such as AP-1, NFAT, CREB and NF-κB [15,16]. Tax also dysregulates cell cycle control and inactivates tumor suppressors such as p53 and Rb [17,18,19]. The Tax protein is composed of 353 amino acids (40 kDa) and contains several domains that mediate interactions with many cellular proteins [20] (Figure 1A). A thorough understanding of the oncogenic process induced by Tax has been elusive, but hijacking of the ubiquitin-proteasome pathway plays an important role as highlighted in this review.

Tax can be localized in different compartments of the cell including the cytosol, nucleus, Golgi apparatus and endoplasmic reticulum (ER) for dedicated functions to benefit virus replication and persistence. Tax activates the transcription factor NF-κB in the cytoplasm and *cis*-Golgi, whereas it regulates viral gene expression in the nucleus. To coordinate these disparate functions, Tax dynamically shuttles between discrete subcellular compartments [21] via a nuclear localization sequence (NLS) and an nuclear export sequence (NES) (Figure 1A) [22,23]. Tax localization can be influenced by specific stimuli such as genotoxic stress which triggers Tax nuclear export [24]. Tax can also be secreted into the extracellular space where cell-free Tax may contribute to inflammation and pathogenesis [25]. The large number of cellular interacting proteins and dynamic localization patterns underlie the profound effects that Tax exerts on cell proliferation and survival and pathogenesis.

Host immune surveillance targets viral antigens, and Tax is one of the principal targets of cytotoxic T cells (CD8^+^ T lymphocytes) [26]. HTLV-1 can evade adaptive immunity by multiple mechanisms to downregulate viral antigens, including Tax, and enter a state of latency. The viral regulatory proteins HTLV-1 basic leucine zipper factor (HBZ), p30 and Rex exert negative effects on Tax activity and/or expression [27,28]. HBZ is encoded by the minus strand of the 3' HTLV-1 LTR and is ubiquitously expressed in ATL cells. Interestingly, HBZ mRNA has cell growth-promoting activities and can enhance proliferation of T cells [29]. HBZ protein counteracts several functions of Tax, including canonical NF-κB activation, thus supporting the notion that HBZ serves as a critical viral factor for latency [30,31]. Mice expressing transgenic HBZ in CD4+ T cells develop lymphomas and systemic inflammation [32], thus raising the possibility that HBZ may play pathogenic roles in HAM/TSP and ATLL.

One of the key cellular targets of Tax is the NF-κB pathway. Tax interacts with specific components of NF-κB pathways to drive the proliferation, survival and transformation of HTLV-1 infected T cells. NF-κB is a family of transcription factors that regulate diverse functions such as immunity, cell cycle, apoptosis, inflammation, development and lymphoid organogenesis [33,34]. The NF-κB family includes NF-κB1/p105, NF-κB2/p100, RelA/p65, RelB and c-Rel. NF-κB forms various combinations of homo- and heterodimers that activate specific genes in a highly directed and regulated manner. NF-κB signaling consists of two distinct pathways with unique biological functions: canonical (also known as classical) and noncanonical (also known as alternative) pathways, regulated by different mechanisms.

NF-κB dimers are retained in the cytoplasm of unstimulated cells by association with IκB inhibitory proteins. Pro-inflammatory cytokines (e.g., TNF and IL-1β), growth factors (e.g., epidermal growth factor receptor), antigen receptors (e.g., T cell receptor) and bacterial lipopolysaccharide (LPS) activate a high molecular weight IKK complex composed of IKKα, IKKβ and IKKγ (also known as NEMO) which phosphorylates IκB proteins on two amino (*N*)-terminal serine residues. Phosphorylation of IκB triggers its ubiquitination and proteasomal degradation to liberate NF-κB complexes which concomitantly translocate into the nucleus to bind and activate specific promoters and gene expression. IKK is directly activated by upstream kinases, most notably the TGF-β activated kinase 1 (TAK1) which phosphorylates key serine residues in the activation loop of IKKβ. TAK1 is recruited to ubiquitinated adaptor molecules in specific pathways (e.g., RIP1 downstream of TNFR1; TRAF6 downstream of IL-1R) via TAB2/TAB3 adaptor molecules bearing ubiquitin-binding domains [35,36]. Similarly, NEMO recruits IKKα/β to these ubiquitinated adaptors through its UBAN (ubiquitin-binding in ABIN and NEMO) domain to facilitate TAK1-induced IKKβ activation [37]. Therefore, NF-κB signaling is tightly regulated by dynamic post-translational modifications (PTMs) such as ubiquitination on key signaling proteins [38,39,40,41].

The noncanonical NF-κB pathway involves stimulus-dependent processing of the NF-κB2 precursor protein to p52, which dimerizes with RelB and regulates expression of genes controlling B cell survival, bone metabolism and lymphoid organogenesis [42]. Various members of the tumor necrosis factor (TNF) superfamily including BAFF, CD40L, RANKL, LT-β and CD27 can trigger activation of the noncanonical pathway [43]. The MAP kinase kinase kinase (MAP3K) member NF-κB inducing kinase (NIK) is a central component of noncanonical signaling and promotes the activation of IKKα, which in turn phosphorylates p100 to trigger its proteasome-dependent processing [44,45,46]. NIK is mainly regulated post-translationally due to constitutive turnover by an E3 ligase complex containing cIAP1, cIAP2, TRAF2 and TRAF3 thus maintaining low levels of NIK protein in most cell types [47,48]. In response to stimulation by specific TNF family ligands, TRAF3 undergoes degradation that inactivates the TRAF/cIAP complex, resulting in stabilization of NIK. The noncanonical NF-κB pathway is aberrantly activated (in the absence of ligand) by somatic mutations in NIK, TRAF2, TRAF3, cIAP1/cIAP2 and other NF-κB regulatory proteins in multiple myeloma [49,50].

Many cancers, including solid tumors and leukemias/lymphomas, exhibit dysregulated and constitutively activated NF-κB activation. Therefore, perhaps not surprisingly ATLL was also found to have persistently activated NF-κB signaling [51]. Tax constitutively activates both canonical and noncanonical pathways of NF-κB [52,53]. Tax activates the canonical and noncanonical pathways of NF-κB by directly interacting with the NEMO subunit of the IKK complex to activate IKK persistently [53,54,55,56]. Tax may also require the contributions of upstream kinases such as TAK1 to activate IKK, although this topic is controversial [57,58]. Tax can interact with TAK1 and TAB2 [57,59,60] to trigger TAK1 activation and downstream JNK and p38 MAP kinase activation [61]. Upon Tax-mediated IKK activation, IKKβ phosphorylates and triggers the degradation of IκBα and IκBβ followed by the nuclear translocation of RelA/p50 dimers [62,63]. NF-κB plays essential roles in HTLV-1-mediated T-cell transformation [64]. Small molecule IKK inhibitors or dominant-negative IκB mutants render CD4^+^ T cells refractory to Tax-mediated transformation [65,66]. Consistent with these findings, expression of a Tax mutant M22 (T130A, L131S) deficient in the activation of IKK and NF-κB is unable to induce cell proliferation and immortalization of T cells [64].

Tax activates the noncanonical pathway of NF-κB by directly triggering p100 processing to p52 by hijacking the NEMO/IKKα complex [53]. Tax induction of p100 processing is partially dependent on beta-transducin repeat-containing protein, a component of the SCF (Skp, Cullin, F-box) E3 ligase complex [67]. Interestingly, Tax does not require NIK for p100 processing [53], although NIK contributes to Tax-mediated IKKα activation and *trans*-activation of NF-κB [68]. IKKα phosphorylates RelA at Ser536 which is critical for its *trans*-activation in response to Tax expression [69]. The noncanonical NF-κB pathway has emerged recently as a critical determinant of Tax-mediated tumorigenesis both *in vitro* and *in vivo* [70,71]. Tax requires a domain between amino acids 225-232 for activation of noncanonical NF-κB and high transforming activity (Figure 1A) [72]. Interestingly, this domain is not conserved in Tax2 encoded by the less pathogenic HTLV-2 [72]. Tax1 also contains a PDZ binding motif (PBM) located in the *C*-terminus (Figure 1A) that is lacking in Tax2 [73]. The Tax PBM mediates interaction of Tax with PDZ-containing proteins, including the tumor suppressor Dlg [74]. Both the PBM and NF-κB2 activation domain appear to play important roles in transformation by Tax [70].

ATL cells exhibit a robust activation of canonical and noncanonical NF-κB despite downregulated or absent Tax expression due to deletions or epigenetic silencing of the 5' LTR or mutations in Tax [75,76]. The mechanisms of Tax-independent chronic activation of NF-κB remain poorly understood but may result from epigenetic alterations. For example, epigenetic downregulation of microRNA-31 (miR-31) in ATLL promotes increased NIK expression and noncanonical NF-κB activation [77]. Whether somatic mutations or other genetic changes play any role in Tax-independent NF-κB activation is unknown.

HTLV-1, as is typical of many viruses, usurps the host cell machinery, including ubiquitination and SUMOylation, to enhance viral replication and evade immune responses. Ubiquitination is a type of PTM, together with phosphorylation, SUMOylation, methylation and glycosylation, that play key regulatory functions for proteins. These modifications are essential for all physiological processes in cells, influence the balance between normal and pathogenic cellular signaling and determine the final outcome of viral infections. Virus infection also generates PTMs that target both cellular and viral proteins. Ultimately, these modifications can lead to enhanced replication of the virus or alternatively an effective host response to eliminate the virus. Ubiquitination is a reversible mechanism whereby ubiquitin molecules are conjugated to a specific protein substrate on lysine (K) residues through a cascade of enzyme reactions. Three types of enzymes act sequentially to link the ubiquitin molecule to the substrate: Ub-activating enzyme (E1), Ub-conjugating enzyme (E2) and Ub-Ligase (E3). The substrates can undergo mono- or polyubiquitination, with either a single or multiple ubiquitin molecules conjugated on the targeted protein respectively. Ubiquitin contains 7 lysine residues (K6, 11, 27, 29, 33, 48 and 63) and each of these can serve as an acceptor for linkage-specific polyubiquitin chain formation. K48-linked polyubiquitin chains serve as signals for protein degradation by targeting to the 26S subunit of the proteasome. K63-linked polyubiquitin chains generally do not trigger protein degradation but rather modulate kinase activation, cell signaling, receptor trafficking and DNA repair [78]. Polyubiquitin chains can also be linked head-to-tail (Met-1), known as linear ubiquitination [79,80]. Linear ubiquitination is carried out by the LUBAC complex consisting of HOIL-1L, HOIP and SHARPIN and plays critical roles in innate immune signaling and certain canonical NF-κB pathways [81].

Ubiquitination is a reversible process and there are approximately 100 deubiquitinating enzymes (DUBs) encoded in the human genome. There are five families of DUBs including ubiquitin *C*-terminal hydrolases (UCHs), ubiquitin-specific proteases (USPs), ovarian tumor proteases (OTUs), Josephins and JAB1/MPN/MOV34 metalloenzymes (JAMMs) [82]. All of these DUBs function as cysteine proteases with the exception of JAMMs which are zinc-dependent metalloproteases [82]. DUBs cleave ubiquitin from target proteins in order to regulate the stability (in case of K48 Ub) or activation (in case of K63 or Met1-Ub) of specific targets [83]. A common characteristic of DUBs is the presence of ubiquitin-binding domains that bind ubiquitin chains on specific target proteins [84].

## 2. Tax and PTMs

### 2.1. Tax Ubiquitination and SUMOylation

In HTLV-1 infected cells Tax is subject to multiple PTMs such as ubiquitination, phosphorylation, SUMOylation and acetylation (Figure 1B). In this review, we shall focus mainly on Tax ubiquitination and SUMOylation. In addition, Tax can induce the modification of host cell proteins in order to modulate their functions and induce pathogenic signaling leading to transformation. Tax PTMs influence Tax cellular localization, *trans*-activation and protein-protein interactions. Tax ubiquitination regulates Tax stability, trafficking and NF-κB activation. Tax has been shown to directly engage proteasomal subunits HsN3 and HC9 to accelerate proteolysis of the NF-κB precursor protein p105 [85,86]. Both mono- and polyubiquitination of Tax were initially reported and proposed to regulate proteasome binding [87]. However, it was clear that Tax ubiquitination did not merely trigger its degradation but also regulated Tax function [88]. Indeed, the mono-ubiquitination of Tax induced by DNA damage on K280 and K284 triggered the export of Tax from the nucleus to the cytoplasm [89]. Conversely, polyubiquitination regulates Tax stability and NF-κB activation. Mutation of key *C*-terminal lysine residues (K280 and K284) impairs Tax ubiquitination and activation of the NF-κB pathway [90]. At steady-state, Tax ubiquitination is largely composed of K63-linked polyubiquitin chains, which regulates NF-κB activation [91]. K63-linked polyubiquitin chains conjugated onto Tax likely serve as molecular platforms for recruitment of kinase complexes such as TAK1 and IKK, analogous to cellular proteins like RIP1. The E2 enzyme Ubc13 is required for the K63-linked polyubiquitination of Tax and knockdown of Ubc13 with short interfering RNA (siRNA) impairs Tax K63-linked polyubiquitination, NEMO/IKK binding and NF-κB activation [91]. Although overexpression of TRAF2, 5 and 6 can potentiate Tax polyubiquitination [60], whether these E3s are necessary for Tax K63-linked polyubiquitination is unclear. It remains unknown which E3 ligase(s) is important for Tax K63-linked polyubiquitination.

**Figure 2:**
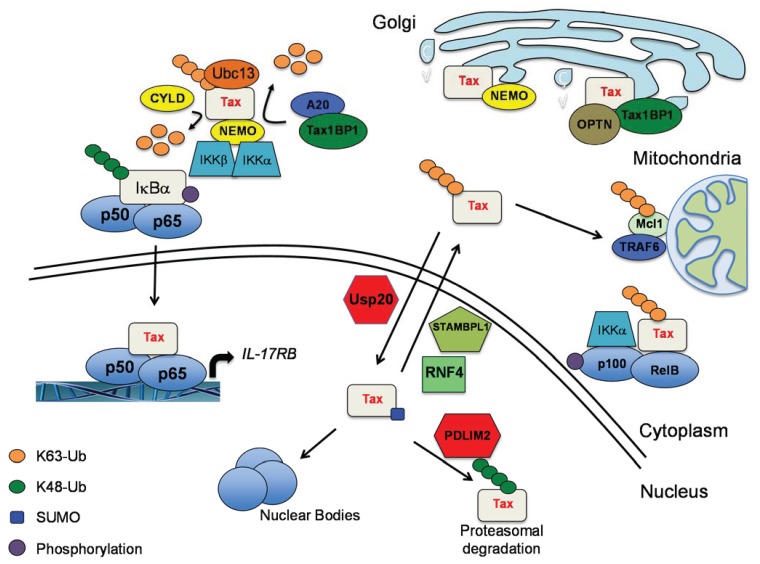
A model depicting Tax-induced shuttling, localization and activation of canonical and noncanonical NF-κB signaling pathways.

Tax K63-linked polyubiquitination may also mediate interactions with additional components involved in NF-κB activation. Tax interacts with NEMO-related protein (NRP)/Optineurin (OPTN) and together form a complex with NEMO to induce sustained NF-κB activation (Figure 2) [92]. The interaction between Tax and OPTN requires Tax ubiquitination sites and the ubiquitin-binding domain of OPTN [92]. Interestingly, the Tax, OPTN and NEMO complex also includes the adaptor molecule TAX1BP1 and is localized to the Golgi [92]. In addition, ubiquitination of Tax on K263, K280 and K284 is required for Tax-induced relocalization of NEMO and the IKK complex to the *cis*-Golgi [93]. Interestingly, Tax has also been shown to activate IKK in Golgi-associated lipid raft microdomains [94]. In addition to the Golgi, Tax-induced IKK activation has also been proposed to occur in or near the centrosome [95]. Taken together, it appears that Tax utilizes the Golgi, and possibly other cytoplasmic substructures, as a specific platform for IKK activation. Tax also promotes the K63-linked polyubiquitination of NEMO; however, this event does not appear to be important for Tax-mediated NF-κB activation [58]. Although there is general agreement that K63-linked polyubiquitination is necessary for Tax-mediated activation of IKK, it appears that linear ubiquitination is dispensable [96]. Whether Tax undergoes polyubiquitination with chains other than K48- and K63-linked is unknown.

Tax also undergoes SUMOylation at sites that overlap the known ubiquitin sites although its role in NF-κB activation remains controversial [97]. SUMOylated Tax associates with p300, RelA and NEMO in nuclear bodies or speckles [98,99]. Tax SUMOylation mainly occurs at K280 and K284, overlapping with the key ubiquitination sites [98]. However, two recent studies using Tax mutants impaired in SUMOylation cast doubt on a role for SUMOylation in Tax-mediated NF-κB activation [100,101].

### 2.2. The Interplay of Tax with Cellular DUBs

Tax ubiquitination can be counteracted by host DUBs that cleave polyubiquitin chains to regulate Tax stability and localization. A recent study showed that CYLD, a DUB and tumor suppressor involved in NF-κB signaling pathways, interacts with Tax and removes ubiquitin chains that results in impaired Tax interaction with NEMO [102]. In HTLV-1 infected cells CYLD is constitutively phosphorylated by IKK, which impairs its function thus allowing persistent NF-κB activation [102,103]. USP20 was also identified as a DUB which opposes Tax ubiquitination and NF-κB activation [104]. USP20 is downregulated in HTLV-1 infected cell lines, presumably to promote high levels of NF-κB activation, although the exact mechanism is unclear [104].

Another important DUB that plays a prominent role in the negative regulation of key NF-κB pathways is A20 (also known as TNFAIP3) [105,106]. A20 requires a ubiquitin-binding adaptor molecule known as TAX1BP1 to engage its substrates RIP1 and TRAF6 for inactivation and signal termination [107,108]. Interestingly, TAX1BP1 was first identified by yeast two-hybrid screening as a binding partner of Tax, A20 and TRAF6 [109,110,111]. In addition to TAX1BP1, A20 is also dependent on other co-factors including the E3 ligases RNF11 and Itch that inducibly assemble to form the A20-ubiquitin-editing complex [112,113]. Cytokine (TNF or IL-1β)-induced mobilization of the A20 ubiquitin-editing complex is triggered by IKKα-mediated phosphorylation of TAX1BP1 [114]. Tax interacts with TAX1BP1 to inactivate the A20 complex by impairing interactions between members of the complex [113]. Tax prevents the assembly of the A20 complex by blocking IKKα-mediated phosphorylation of TAX1BP1 [114]. A20 can also block NF-κB by disrupting E2:E3 enzyme interactions and concomitant degradation of the E2 enzyme Ubc13, however Tax opposes this function of A20 to preserve Tax K63-linked polyubiquitination and NF-κB activation [115]. Tax inactivation of A20 alleviates the negative feedback control of NF-κB and facilitates persistent NF-κB signaling that drives cell transformation. A recent study has demonstrated that Tax promotes autophagy via IKK activation that supports the proliferation and survival of HTLV-1 transformed cell lines [116]. Given that TAX1BP1 may also function as an autophagy receptor [117], it is plausible that Tax may have also hijacked the autophagy function of TAX1BP1 for cell transformation.

Tax contains NLS and NES sequences which regulate the nucleo-cytoplasmic shuttling of Tax. A fine regulation of Tax cellular distribution is controlled by specific PTMs, leading to conformational changes and inducible protein-protein interactions. The balance between Tax ubiquitination and SUMOylation may potentially regulate Tax shuttling between different cellular compartments and activation of NF-κB [20]. The E3 ligase PDLIM2 conjugates Tax with K48-linked polyubiquitin chains to trigger Tax proteasomal degradation in the nuclear matrix [118]. However, Tax interaction with HSP90 protects Tax from proteasomal degradation in the nuclear matrix [119,120]. We recently conducted an RNAi library screen for DUBs that regulate Tax-mediated NF-κB activation. This endeavor led to the identification of the metalloprotease STAM-binding protein like 1 (STAMBPL1) as a DUB regulating Tax cellular localization [121]. STAMBPL1 indirectly regulated Tax activation of NF-κB by promoting Tax nuclear export [121]. However, STAMBPL1 did not directly cleave ubiquitin molecules from Tax, and the precise targets and substrates of STAMBPL1 remain unknown. Another study identified the SUMO-targeted E3 ligase RNF4 (Really Interesting New gene Finger protein 4) as a new binding partner of Tax and regulator of Tax shuttling [122]. RNF4 ubiquitinates Tax and promotes its nuclear export to enhance NF-κB activation [122]. It is possible that RNF4 may function together with STAMBPL1 to facilitate Tax nuclear export.

### 2.3. Proteins Ubiquitinated Downstream of Tax

Tax can promote PTMs of a multitude of host cell proteins, including the IKK complex, in the step-wise progression of cellular transformation. It has been demonstrated that Tax interacts with all three components of the IKK complex, however its interaction with NEMO is requisite for the modification and activation of the IKK catalytic subunits [123]. Tax binds to and triggers NEMO oligomerization to enhance IKK activation [124]. Upon IKKβ phosphorylation of Ser 177/181, the kinase is mono-ubiquitinated which is required for the activation of the IKK complex [125]. Tax likely sustains IKK phosphorylation by inhibition of protein phosphatase 2A (PP2A) [126]. The cumulative Tax-induced modifications of the IKK complex induce downstream phosphorylation and proteasomal degradation of IκBα and IκBβ [55,62]. In addition to IKK, a number of other proteins are modified by ubiquitin downstream of Tax. Tax interacts with FoxO4, a tumor suppressor protein, and the E3 ligase MDM2 and triggers the ubiquitination and degradation of FOXO4 by the proteasome [127]. Another protein targeted for degradation by Tax is the tumor suppressor Rb [19]. Finally, Tax dysregulates cell cycle control and establishes mitotic abnormalities by activating the APC^CDC20^ E3 ligase complex ahead of schedule leading to proteasomal degradation of cyclin B1 and securin [128].

To identify proteins downstream of Tax that are ubiquitinated, we recently undertook an unbiased proteome-wide approach using mass spectrometry. Jurkat cells expressing tetracycline-inducible wild-type Tax or Tax M22 were subjected to a ubiquitin proteomics screen using a ubiquitin branch (K-ε-GG) antibody to enrich ubiquitinated peptides for liquid chromatography-tandem mass spectrometry (LC-MS/MS). We found that Tax induced the ubiquitination of 136 proteins, and 22 of these were dependent on IKK activity (induced by wild-type Tax but not M22) [129]. One of these was the antiapoptotic Bcl-2 family member MCL-1 which undergoes K63-linked polyubiquitination in response to Tax expression. Tax-induced MCL-1 ubiquitination was dependent on the E3 ligase TRAF6 and the IKK complex. Tax contains a TRAF6 interacting motif just upstream of the PBM in the *C*-terminus (Figure 1A). Tax binds to, activates TRAF6 and induces its mitochondrial localization where it conjugates MCL-1 with K63-linked polyubiquitin chains to stabilize and protect it from degradation induced by genotoxic stress (Figure 2) [129]. Both TRAF6 and MCL-1 are essential for HTLV-1-induced immortalization of primary T cells [129]. Although TRAF6 was dispensable for Tax to activate IKK in an *in vitro* system [96], we have found that TRAF6 indeed plays an important role in NF-κB activation in HTLV-1 transformed T cell lines [130]. It remains to be determined if the *C*-terminal TRAF6 interaction motif in Tax is important for Tax to activate IKK and NF-κB.

To gain more insight into the mechanisms of HTLV-1 T-cell transformation, we recently conducted a next-generation RNA sequencing study to identify genes aberrantly expressed in T cells immortalized by HTLV-1. This effort identified the IL-25 receptor subunit IL-17RB as an aberrantly overexpressed gene in HTLV-1 immortalized T cells [130]. Tax induced the expression of IL-17RB in an IKK and NF-κB-dependent manner (Figure 2), and IL-17RB was essential for HTLV-1-induced T-cell immortalization and the proliferation and survival of HTLV-1 transformed T cell lines [130]. Because IL-17RB signaling activates TRAF6 [131], it is conceivable that Tax may synergize with the IL-17RB pathway to activate TRAF6 for NF-κB activation and MCL-1 stabilization. Our cumulative results support a model whereby Tax generates an IL-17RB-NF-κB feed-forward autocrine loop that is obligatory for HTLV-1 leukemogenesis. Interestingly, IL-17RB is overexpressed in leukemic cells from ATLL patients and also supports NF-κB signaling in a subset of Tax-negative ATLL cell lines [130]. IL-17RB is encoded on chromosome 3p21.1, a region that is frequently amplified in aggressive acute ATLL cases [132]. Therefore, amplification of the *IL-17RB* gene may potentially compensate for the loss of Tax-induced NF-κB in ATLL. It is also plausible that distinct receptors may be amplified or overexpressed by other mechanisms to drive NF-κB signaling in IL-17RB-negative ATLL cases.

## 3. Conclusions

In this review, we have highlighted some of the mechanisms used by the multifunctional HTLV-1 Tax oncoprotein to hijack the cellular ubiquitin-proteasome machinery to promote aberrant signaling linked to cell survival and proliferation. Ubiquitination serves as a versatile tool that can modulate Tax function including nuclear export (mono-ubiquitination), degradation in the nuclear matrix (K48-linked polyubiquitination) or NEMO binding and IKK/NF-κB activation (K63-linked polyubiquitination). Tax hijacks the host ubiquitin machinery to promote its K63-linked polyubiquitination, likely to enhance NEMO binding and other protein-protein interactions in the *cis*-Golgi where Tax activates IKK [91,93]. Interestingly, Tax2 encoded by HTLV-2 does not undergo detectable ubiquitination, yet still activates NF-κB [133]. These results underscore fundamental differences by which Tax1 and Tax2 activate NF-κB.

Tax also activates host E3 ligases (TRAF6) to stabilize MCL-1 and mitigate cell death triggered by genotoxic stress agents and chemotherapy drugs such as etoposide [129]. Conversely, Tax inactivates multiple DUBs that oppose NF-κB activation including A20 and CYLD [113]. Although Tax has usurped the host ubiquitin-proteasome pathway for NF-κB activation and cell transformation, there is much still to be learned regarding the precise mechanisms. We do not know the identity of the K63-Ub specific E3 for Tax or potential roles for other E2s and/or E3s. In this regard, mass-spectrometry based proteomic screens or yeast two-hybrid screens may have utility to identify ubiquitin-proteasome components that interact with Tax. In addition, screening of E2 and E3 enzyme siRNA libraries may identify key ubiquitin-proteasome components that regulate Tax function, stability and/or trafficking. Delineation of the complex interplay between Tax and the host ubiquitin-proteasome machinery may yield novel drug targets for HTLV-1-associated diseases.
